# Impact of Zirconia Three‐Unit Resin‐Bonded Fixed Partial Dentures in the Posterior Region on Oral Health‐Related Quality of Life: A 12‐Month Longitudinal Observation

**DOI:** 10.1111/jerd.70122

**Published:** 2026-02-12

**Authors:** Rebecca Handermann, Andreas Zenthöfer, Brigitte Ohlmann, Moritz Waldecker, Peter Rammelsberg, Wolfgang Bömicke

**Affiliations:** ^1^ Department of Prosthodontics Heidelberg University Hospital, Heidelberg University Heidelberg Germany

**Keywords:** ceramic restorations, dPrOM, fixed dental prothesis, minimal‐invasive dentistry, OHIP‐14, OHRQoL, RBFPDS

## Abstract

**Introduction:**

Patient‐reported outcome measures (PROMs) remain limited in prosthodontics, including for ceramic resin‐bonded fixed partial dentures (RBFPDs), despite their growing relevance in minimally invasive practice. Oral health–related quality of life (OHRQoL) can be reliably assessed using the validated Oral Health Impact Profile −14 (OHIP‐14) questionnaire.

**Methods:**

Thirty participants completed the German version of the OHIP‐14 before and 3 months as well as 12 months after receiving a three‐unit posterior monolithic zirconia RBFPD (*n* = 30). Scores were summarized descriptively; clinical relevance was assessed using effect size and the Minimal Important Difference (MID). Longitudinal differences were examined using the Wilcoxon signed‐rank test (*α* = 0.05).

**Results:**

Mean total scores decreased from 4.2 ± 3.9 at baseline to 1.8 ± 2.9 at 12 months. The baseline‐to‐12‐month difference (−2.38) exceeded the two‐point MID. Significant improvements occurred from baseline to 12 months (*p* < 0.001), accompanied by a moderate effect size between baseline and 12 months (d = −0.59). After 12 months, significant improvements were found in the domains orofacial pain (*p* = 0.010) and orofacial appearance (*p* = 0.035).

**Conclusion:**

Serving participants posterior three‐unit zirconia RBFPDs led to clinically meaningful OHIP‐14 improvements, with significant gains in the domains of orofacial pain and orofacial appearance.

**Clinical Significance:**

These findings highlight the positive impact of zirconia RBFPDs on OHRQoL when used for posterior tooth replacement.

## 1 Introduction

The clinical success of dental restorations depends on multiple factors, and its assessment should extend beyond technical and biological prognostic parameters [1, 2]. Tooth loss can impair chewing, smiling, and speaking, thereby reducing oral health–related quality of life (OHRQoL) [3, 4, 5, 6, 7, 8]. The degree of impairment shows considerable interindividual variation, and even a single missing tooth may result in substantial functional limitations [9, 10]. Consequently, prosthetic treatment should be patient‐centered and tailored to individual preferences [11, 12]. Shared decision‐making (SDM) promotes transparent communication and has been shown to enhance satisfaction, adherence, and the dentist–patient relationship while reducing treatment errors and revisions [13, 14, 15, 16, 17, 18]. The Oral Health Impact Profile (OHIP) is a validated dental patient‐reported outcome measure (dPROM) grounded in the WHO model of impairments, disabilities, and handicaps and conceptually organized into four clinically meaningful domains: oral function [19], orofacial pain [20], orofacial appearance [21], and psychosocial impact [1, 8, 22, 23, 24, 25, 26, 27, 28, 29, 30, 31, 32, 33, 34, 35]. Studies using OHIP scores consistently demonstrate significant improvements in OHRQoL after dental interventions, for example following single‐tooth replacement with fixed or removable dental prostheses or single‐tooth implants [36, 37, 38, 39]. However, SDM in dental practice should extend beyond the mere selection of treatment type and also encompass the choice of appropriate restorative materials. In this context, monolithic zirconia has gained particular attention, as it is increasingly being evaluated for various prosthetic restorations, including resin‐bonded fixed partial dentures (RBFPDs). Excellent long‐term clinical survival data are available for metal–ceramic RBFPDs [40, 41, 42, 43, 44], along with strong evidence of favorable patient‐reported OHRQoL [43, 44, 45, 46, 47, 48]. Promising initial clinical results have also been reported for ceramic RBFPDs in the posterior region [40, 41]. A recent prospective clinical study on posterior cantilevered zirconia RBFPDs found significant improvements in OHIP‐49 scores 3 years after treatment [46]. However, data on patient perspectives on ceramic RBFPDs in this context remain scarce. The present study seeks to augment the available evidence by evaluating mid‐term changes in OHRQoL among patients treated with monolithic zirconia three‐unit RBFPDs in the posterior region using *p*‐values, effect size (ES) [49, 50], and the minimal important difference (MID) [30, 34]. The primary objective was to quantify patient‐reported changes between baseline (before treatment) and 3‐ and 12‐month follow‐ups through OHIP‐14 summary scores [30, 34]. The study hypothesis was that OHRQoL would remain unchanged following RBFPD therapy.

## 2 Material and Methods

The local ethics committee approved the study protocol (registration no. S‐083/2013), and it was registered in advance at clinicaltrials.gov (registration no. NCT01997710). Patients presenting for treatment at the Department of Prosthodontics at Heidelberg University Hospital were considered eligible if they required prosthetic replacement of at least one missing second premolar, first molar, or second molar with fixed antagonists. Additional inclusion criteria were age between 18 and 89 years, good oral hygiene, and meeting the indication for treatment with an RBFPD in the posterior region [40]. All participants provided written informed consent and agreed to attend regular follow‐up visits. Exclusion criteria included systemic or mental health conditions preventing standard dental treatment, inability to provide informed consent, pregnancy, breastfeeding, or substance abuse. The data analyzed in this study were obtained as a secondary outcome measure from a randomized controlled trial evaluating the prognostic performance of zirconia RBFPDs in 30 participants. No a priori sample size calculation was performed for the present analysis, as OHRQoL was defined as secondary endpoint within a pilot clinical trial. Each participant received a single RBFPD. All RBFPDs were fabricated as monolithic restorations using 3 mol% yttria‐stabilized tetragonal zirconia polycrystal (Cercon ht light/medium, DeguDent GmbH), and according to randomization, were retained either by inlays or by wings. For the present evaluation, OHIP data from participants with both RBFPD designs were pooled for analysis [40, 41].

Participants completed the German version of the OHIP‐14 (OHIP‐G14) questionnaire prior to receiving RBFPDs, as well as at 3 months and 12 months posttreatment [27, 32, 33]. The questionnaire comprises 14 items, each rated on a five‐point Likert scale (4 = very often, 3 = often, 2 = occasionally, 1 = rarely, 0 = never), all referring to experiences during the previous month. Thus, total scores could range from 0 to 56, with lower scores indicating better OHRQoL. The questionnaires were checked for completeness immediately after completion, and patients were asked to answer any missing items. Responses were summarized for baseline, 3‐month, and 1‐year follow‐ups using descriptive statistics, including mean, median, standard deviation, minimum and maximum values, as well as the 25th and 75th percentiles. Additionally, a four‐dimensional characterization of OHRQoL in (1) oral function, (2) orofacial pain, (3) orofacial appearance, and (4) psychosocial impact was performed using summary scores of the corresponding OHIP‐14 items [28]. Specifically, questions 7 and 8 (“Diet unsatisfactory” and “Interrupt meals”) reflect overall oral function, questions 3 and 4 (“Painful aching” and “uncomfortable to eat”) focus on orofacial pain, questions 5 and 6 (“tense” and “self‐conscious”) assess orofacial appearance, and questions 13 and 14 (“Life unsatisfying” and “unable to function”) address psychosocial impact [28]. Pairwise comparisons of total summary and dimensional summary scores between the three time points (baseline vs. 3 months, baseline vs. 12 months, and 3 months vs. 12 months) were performed using the Wilcoxon test. Based on the means and standard deviations of the score differences between measurement times, pairwise ES [49] were calculated for both the total and the dimensional scores [28, 30, 34, 35]. Additionally, MIDs defined as more than 2 points for OHIP‐14 [30, 34] were applied to determine whether the observed changes in summary scores were clinically meaningful. All analyses were performed with SPSS software, version 27. Statistical significance was set at *p* = 0.05.

## 3 Results

Sociodemographic and restoration characteristics of the study participants at baseline are presented in Table 1. Fully completed questionnaires were available for 30 participants at baseline and at the 3‐month follow‐up. At the 12‐month follow‐up, data were available for 29 participants due to failure of one RBFPD in the interim.

**TABLE 1:** Sociodemographic and restoration characteristics of participants (*n* = 30) at baseline.

Variable	Outcome	
RBFPD design, *n* (%)	Inlay‐retained	15 (50)
	Wing‐retained	15 (50)
Mean participant age ± SD (range), years	51.07 ± 13.76 (26.5–75.33)	
Participant sex, *n* (%)	Women	13 (43.3)
	Men	17 (56.7)
Jaw, *n* (%)	Upper Jaw	16 (53.3)
	Lower Jaw	14 (46.7)
RBFPD location, *n* (%)	Second premolar	7 (23.3)
	First molar	20 (66.7)
	Second molar	3 (10)
Mean number of remaining/restored teeth (including wisdom teeth) before RBFPD treatment ± SD (range)	27.37 ± 1.71 (24–30)	
Eichner index, *n* (%)^a^	A1	26 (86.7)
	A3	3 (10)
	B1	1 (3.3)

Abbreviations: RBFPDs = Resin‐bonded fixed partial dentures, ± SD = standard deviation, Eichner index = classification of functional posterior occlusal support [51].

^a^ Categories without entries omitted for clarity.

Total summary and dimensional summary scores are listed in Table 2. The mean total summary scores were 4.2 ± 3.9 (range 0–17) at baseline, 2.3 ± 3.2 (range 0–15) at the 3‐month recall, and 1.83 ± 2.9 (0–11) at the 12‐month recall.

**TABLE 2:** Descriptive data from OHIP‐14 total and dimensional summary scores at three time points of measurement (baseline, 3‐months follow‐up and 12‐months follow‐up).

Scores		*N*	Mean	Median	SD±	Range	25th pct	75th pct
Total summary score								
	Baseline	30	4.20	3.00	3.87	0–17	1.00	6.25
	3 Months		2.30	0.00	3.23	0–15	0.00	3.00
	12 Months	29	1.83	0.00	2.89	0–11	0.00	3.00
Domaine summary score								
Psychosocial impact	Baseline	30	0.30	0.00	0.47	0–1	0.00	1.00
	3 Months		0.17	0.00	0.6	0–3	0.00	0.00
	12 Months	29	0.21	0.00	0.77	0–4	0.00	0.00
Oral function	Baseline	30	0.40	0.00	0.86	0–4	0.00	1.00
	3 Months		0.20	0.00	0.55	0–2	0.00	0.00
	12 Months	29	0.14	0.00	0.35	0–1	0.00	0.00
Orofacial appearance	Baseline	30	0.97	0.50	1.30	0–5	0.00	2.00
	3 Months		0.21	0.00	0.49	0–2	0.00	0.00
	12 Months	29	0.28	0.00	0.65	0–2	0.00	0.00
Orofacial pain	Baseline	30	1.37	1.00	1.27	0–4	0.00	2.00
	3 Months		0.87	0.00	1.11	0–4	0.00	2.00
	12 Months	29	0.62	0.00	1.08	0–4	0.00	1.00

Abbrevaitions: 25th pct = 25. percentile, 75th pct = 75. percentile, *N* = number of participants, SD± = standard deviation.

For orofacial appearance, statistically significant differences were found between baseline and 3 months (*p* = 0.003) and between baseline and 12 months (*p* = 0.035) (Table 3). In the domain orofacial pain, a statistically significant difference was found between baseline and 12 months (*p* = 0.010). No statistically significant differences were observed in any pairwise comparison of the three time points for the psychosocial impact or oral function domains.

**TABLE 3:** OHIP‐14 Summary score differences for the total score and dimensional scores between the three time points of measurement (baseline, 3‐months follow‐up and 12‐months follow‐up).

Pairwise scores differences		*N*	Mean	Median	SD±	Range	25th pct	75th pct	ES	*p*
Total summary score										
	Baseline versus 3 months	30	−1.97	−1.00	3.14	−10–4	−4	0.00	−0.62	0.002^a^
	3 Months versus 12 months	29	−0.45	0.00	3.02	−7–9	−1.00	0.00	−0.15	0.186
	12 Months versus baseline	29	−2.38	−2	4.01	−14–8	−4.00	0.00	−0.59	0.001^a^
Domaine summary score										
Psychosocial impact	Baseline versus 3 months	30	−0.13	0.00	0.78	−1–3	−1.00	0.00	−0.17	0.166
	3 Months versus 12 months	29	0.07	0.00	0.37	−1–1	0.00	0.00	−0.19	0.317
	12 Months versus baseline	29	−0.10	0.00	0.94	−1–4	−1.00	0.00	−0.11	0.166
Oral function	Baseline versus 3 months	30	−0.20	0.00	1.03	−4–2	−0.25	0.00	−0.19	0.353
	3 Months versus 12 months	29	−0.07	0.00	0.65	−2–1	0.00	0.00	−0.11	0.516
	12 Months versus baseline	29	−0.24	0.00	0.830	−3–1	−0.50	0.00	−0.29	0.131
Orofacial appearance	Baseline versus 3 months	30	−0.72	0.00	1.19	−5–1	−1.5	0.00	−0.61	0.003^a^
	3 Months versus 12 months	28	0.07	0.00	0.716	−2–2	0.00	0.00	0.01	0.581
	12 Months versus baseline	29	−0.66	0.00	1.50	−5–2	−1.5	0.00	−0.44	0.035^a^
Orofacial pain	Baseline versus 3 months	30	−0.50	0.00	1.53	−4–3	−1.25	0.00	−0.33	0.109
	3 Months versus 12 months	29	−0.21	0.00	1.21	−3–3	−0.50	0.00	−0.17	0.343
	12 Months versus baseline	29	−0.76	0.00	1.41	−4–2	−2.00	0.00	−0.54	0.010^a^

Abbreviations: 25th pct = 25. percentile, 75th pct = 75. percentile, ES = effect size, *N* = number of participants, *p* = *p*‐value, SD ± = standard deviation.

^a^ Beyond significance level set at 0.05.

The Wilcoxon test revealed a statistically significant difference between the total summary scores at baseline and 3 months (*p* = 0.002), as well as between baseline and 12 months (*p* < 0.001). No significant difference was observed between the 3‐month and 12‐month recall scores (*p* = 0.186) (Table 3).

MID showed improvements in 15 participants (51.7%) between baseline and 3 months, while five participants (17.2%) demonstrated improvement between 3 months and 12 months, and 12 participants (43.3%) showed improvement between baseline and 12 months (Table 4).

**TABLE 4:** Distribution of minimal important difference.

Comparison	Deterioration (%)	No change (%)	Improvement (%)	Total participants (*n*)
Baseline versus 3 months	1 (3.4)	13 (44.8)	15 (51.7)	29
3 Months versus 12 months	3 (10.3)	21 (72.4)	5 (17.2)	29
12 Months versus baseline	0 (0)	17 (56.7)	12 (43.3)	30

*Note:* Minimal important difference corresponding to 2 OHIP‐points for OHIP‐14 [35].

A moderate ES was observed between the baseline and 12‐month measurements (*d* = −0.59) [50]. Similarly, the change from baseline to 3 months showed a comparable effect magnitude (*d* = −0.63). The ES between the 3‐month and 12‐month recall was small (*d* = −0.15) (Table 3).

## 4 Discussion

The findings of the present study demonstrated a marked improvement in the patients' OHRQoL condition following RBFPD treatment, which persisted throughout the follow‐up period of 1 year. A significant improvement in OHRQoL was observed at 3 and 12 months following the placement of a posterior three‐unit zirconia RBFPD, with statistically significant *p*‐values and notable ES [50]. The study hypothesis was therefore rejected. Since the mean difference between baseline and 12 months exceeded the minimally important difference (MID), the observed change in OHRQoL could be considered clinically relevant [34].

Based on normative OHIP‐14 values reported by John et al. [30] the study sample showed moderate oral health‐related impairment at baseline, corresponding approximately to the 70th percentile of the general population [31]. After 3 and 12 months, mean OHIP‐14 scores slightly undercut the population median, indicating an improvement of OHRQoL after fitting of the zirconia RBFPDs as well. When comparing the present mean scores with findings from other studies evaluating OHRQoL after dental treatments using the OHIP‐14 questionnaire, one study on single‐tooth replacement with either an implant or a tooth‐supported fixed dental prosthesis reported larger mean score changes of −4.4 and −4.0, respectively, but also found higher pretreatment mean scores (8.7–10.1 OHIP‐14 points) [9]. Another observational study using the OHIP‐14 questionnaire found higher mean scores of 5.4 before and 3.2 after treatment with fixed and removable partial dentures [38]. In that study, total summary baseline scores were not reported; however, given the types of prosthetic treatments provided, it is likely that participants were missing more teeth than in the present study, and therefore exhibited a higher baseline burden and due to the more extensive dental treatment, greater impact [4]. Nevertheless, significant differences between measurement points and moderate ES in this study were consistent with previous studies assessing OHRQoL after treatment with fixed dental and implant‐supported partial dentures [7, 36, 37, 39] as well as with studies using the OHIP‐49 in patients rehabilitated with metal‐ceramic [45, 48] and ceramic RBFPDs [46].

When analyzing the impact of RBFPD treatment on the four dimensions that cover the concept of OHRQoL, two domains—orofacial appearance and orofacial pain—showed significant differences between baseline and 12 months. Accordingly, these two domains showed the largest mean differences across the three measurement points. The orofacial appearance domain showed a median score of 1 (mean 0.97) at baseline, which decreased to 0 (mean 0.28) at the 12‐month recall. Compared with normative median values ranging from 0.13 (tooth wear) to 3.04 (tooth whitening populations) [21], this indicated that participants in the present study exhibited low baseline impairment and a further improvement over time, approaching values observed in populations without esthetic treatment needs. This was consistent with the fact that the missing teeth were located in the posterior, nonesthetic region.

The mean baseline summary score for orofacial pain decreased from 1.37 to 0.62 OHIP‐14 points. In comparison, normative values for orofacial pain typically range between 2 and 3 OHIP‐14 points, indicating that the values observed in this study were substantially lower than those reported for common orofacial pain conditions (odontogenic pain, oral mucosal pain/burning mouth syndrome, third molar extractions, or temporomandibular disorders) [20]. This outcome was not unexpected, given that only a single missing tooth was involved. Considering the established association between tooth loss and OHRQoL [3, 4, 5] and between OHRQoL and pain [6], the significant reduction in pain perception exhibited in this study may be explained by several factors. Enhanced perceived occlusal stability, reduced compensatory function such as unilateral chewing [9, 10], and the psychological relief associated with closing the single‐tooth gap may have contributed to the observed improvements in pain‐related OHRQoL [6, 48].

The other domains psychosocial impact and oral function already showed baseline values below 0.5, which further decreased slightly over the observation period (−0.1 and −0.24 respectively), although this change was not statistically significant. These values are markedly lower than the normative scores reported in the literature for psychosocial impact (3.2–0.8) [8] and oral function (7.9–0) [19]. This finding is not surprising, as the psychosocial and functional burden associated with a posterior single‐tooth gap—particularly in the presence of four remaining posterior support units—is considered low [4]. Nonetheless, the slight numerical decrease after treatment with zirconia RBFPDs aligned with reports indicating that unilateral tooth loss may still lead to measurable functional limitations and psychosocial consequences, such as reduced ease of speaking or smiling [9, 10]. In contrast to these findings, one similar study [46] reported significant improvements in psychosocial impact and orofacial appearance, but not in orofacial pain, in their prospective 3‐year study with a comparable sample size evaluating ceramic RBFPDs in the posterior region. Because that study used the OHIP‐49, baseline burden is difficult to compare directly. However, given the low baseline burden in the present cohort, the absence of significant differences in the present study may also be attributable to the small sample size.

This study also had its limitations. Summary scores of the OHIP‐14 items show a strong correlation with the summery scores of the OHIP‐5, OHIP‐19, and OHIP‐49 versions [28], and the corresponding OHIP domain scores equally allow for a four‐dimensional characterization of OHRQoL [22, 23, 24, 25, 26, 27, 28]. Hence, recent publications have suggested that even shorter versions, such as the OHIP‐5, can adequately capture the four‐dimensional construct while requiring significantly less time and fewer resources [22, 24, 28]. However, longer versions are recommended when more detailed information on specific impairments—such as the replacement of a single missing tooth, which may relate to a low baseline burden—is desired. It should further be noted that *p*‐values do not reflect the magnitude of change and are strongly influenced by sample size. Given the small sample of 30 participants in this study, clinically meaningful differences may therefore have failed to reach statistical significance [30] due to limited statistical power. Consequently, the results should be interpreted with caution, and their generalizability may be limited. As this analysis represented a secondary endpoint of a clinical pilot study evaluating the prognostic performance of three‐unit zirconia RBFPDs, no a priori sample size calculation was performed. Despite these limitations, the observed MID values and effect sizes support the internal consistency and clinical relevance of the findings. Larger clinical studies are required to evaluate OHRQoL following treatment with zirconia RBFPDs and other restorative options, with the present data informing trial design and sample size determination for single‐tooth replacement.

## 5 Conclusion

Within the limitations of this current study, it was conducted that

1. Treatment with posterior three‐unit zirconia RBFPDs resulted in a notable improvement in patients' OHRQoL that remained stable throughout the 1‐year follow‐up.
2. Despite the low baseline burden, significant reductions in OHIP‐14 summary scores were observed in the domains in which improvement would be expected for posterior single‐tooth replacements—namely, orofacial appearance and orofacial pain.

## Funding

This was an investigator‐initiated study financially supported by DeguDent GmbH. The company had no role in the study design, data collection, analysis or interpretation, manuscript preparation, or the decision to submit the article for publication.

## Disclosure

The authors have nothing to report.

## Conflicts of Interest

The authors declare no conflicts of interest.

## Data Availability

The data that support the findings of this study are available on request from the corresponding author. The data are not publicly available due to privacy or ethical restrictions.
